# Assessment of Anterior Loop of Inferior Alveolar Nerve and Its Anatomic Variations with Age, Gender, and Dentition Status in Indian Population: A CBCT Study

**DOI:** 10.1155/2021/1813603

**Published:** 2021-08-31

**Authors:** Anjali Gupta, Sandeep Kumar, Siddharth Kumar Singh, Arunoday Kumar, Abhishek Gupta, Palkin Mehta

**Affiliations:** ^1^Department of Dentistry, Saraswati Medical College, Unnao, Uttarpradesh 209859, India; ^2^Department of Public Health Dentistry, Dental Institute, Rajendra Institute of Medical Sciences, Ranchi, Jharkhand 834009, India; ^3^Department of Oral Medicine and Radiology, Saraswati Dental College, Lucknow 227105, India; ^4^Department of Prosthodontics and Crown and Bridge, Dental College, Regional Institute of Medical Sciences, Imphal, Manipur 795004, India; ^5^Department of Oral Medicine and Radiology, Chitwan Medical College, Bharatpur 44207, Nepal

## Abstract

**Background:**

The posterior region of the mandible is more often related to iatrogenic errors, but the interforaminal region is also not spared for neurovascular complications. This study aimed to use CBCT images to evaluate the prevalence of anterior nerve looping and its variations with age, gender, and dentition status.

**Methods:**

This retrospective study was carried out by studying 600 CBCT scans retrieved from archival records of a CBCT center in Lucknow. The scans were inspected by two trained investigators. The length of the anterior loop was measured using the measuring tool of Carestream 3D imaging software. Descriptive and analytical tests were performed.

**Results:**

The prevalence of the anterior loop of the inferior alveolar nerve was found to be 56%. The prevalence was found to be more on the right side (29.0%) compared to the left side (27.0%). The most common anterior looping of the inferior alveolar nerve was type 3 followed by type 1. Males were found to have significantly higher loops compared to females. The number of loops was found to decrease significantly with age. The mean length of the loop was found to vary from 1.14 to 1.61 mm.

**Conclusion:**

The anterior looping of IAN is very much prevalent in the Lucknow population. The use of the CBCT technique and appropriate preplanning prior to surgery or implant placement should be performed to prevent nerve injury.

## 1. Introduction

The fifth cranial nerve is known as the trigeminal nerve and has three branches which are the ophthalmic, maxillary, and mandibular. The third branch is called the mandibular nerve. It is the largest of the three branches and carries both afferent and efferent fibers. The mandibular nerve innervates the lower face and the mandible, including the teeth, the temporomandibular joint, and the mucous membrane of the mouth and the anterior two-thirds of the tongue, the mastication muscles, and some smaller muscles [1–3].

The inferior alveolar nerve is a mandibular nerve branch. The inferior alveolar nerve may extend beyond the mental foramen in an anterior and inferior direction, curving back to the foramen and forming a loop, which has been termed the anterior loop of the inferior alveolar nerve, of the mental nerve, or the mandibular canal [4–6]. The prevalence of the inferior alveolar nerve anterior loop has been found to be highly variable ranging from 22% to 94% [7, 8]. In addition, the length of the inferior alveolar nerve anterior loop has shown variations up to a maximum of 11 mm [9]. Some variations in the prevalence of the inferior alveolar nerve anterior loop also exist between different populations and ethnic groups [10].

The evaluation and assessment of the loop are best carried out using the radiographic technique. Radiography provides the clinician with information not readily available by any other diagnostic method. However, the ability of conventional two-dimensional radiological methods (panoramic tomography and periapical radiographs) to reveal the anterior loop is limited, and their reliability and accuracy are questioned [11–13]. Cone-beam computed tomography (CBCT) is a relatively new imaging modality that provides multiplanar views of the facial skeleton with a reduced radiation dose, compared to the most commonly used by MDCT, exposure protocols [14, 15]. MRI has several advantages over other modalities other than the long scan time [16]. CBCT has been used for the identification of the anterior loop. The proposed advantage of these techniques is their ability to create an accurate three-dimensional representation of the structure under investigation, thus eliminating the error of image distortion inherent in plain film radiography. A number of authors have compared CBCT measurements to anatomical dissection and superimposition with soft structures photographed, making its use, aesthetic, and surgical planning easier, and they have concluded that CBCT confers a high degree of accuracy when assessing the presence of an anterior loop [17–19].

For carrying out various surgical procedures and for the placement of implants, it is essential to have a good knowledge of anatomical landmarks in order to avoid complications. Limited studies have been carried out to assess the variations of the anterior loop in Indian populations using advanced radiographic techniques. The aim of this study was to use CBCT images to evaluate the prevalence of anterior nerve looping and compare the mean values of anterior extension of the alveolar loop on right and left sides according to age, gender, and dentition status. The study findings will enable surgical implantologists to accurately locate the course of the inferior alveolar nerve thereby preventing iatrogenic injuries to the nerve.

## 2. Materials and Methods

The retrospective study was conducted using CBCT images from the archives of a CBCT center in Lucknow, U.P. The study was carried out for a duration of 6 months from October 2020–March 2021, wherein retrospective examinations of CBCT scans were carried out. The images of 600 subjects (who had undergone CBCT examination showing the mandibular premolar/molar region) were retrieved from the archival records and inspected for the presence of the mental nerve loop. For the calculation of sample size, a pilot study was done, and based upon the findings of the pilot study, it was decided to retrospectively examine a total of 600 scans which would suffice study objectives. The scans of 600 individuals were bilaterally divided into right and left sides using the advanced software for further data analysis and evaluation. Thus, a total of 1200 sides (right and left) were obtained from 600 scans. A total of nearly 20–25 CBCT scans were examined per day.

The CBCT scans having dentition at the mandibular premolar region and the anterior part at least 2 cm mesial to the mental foramen were included in the study, while scans in which there were any developmental anomalies, pathological lesions, inadequate diagnostic quality, and any history of trauma to the mandible were not included in the study. In the age group between 20 and 80 y, both male and female patients were included in the study. A first pilot study was conducted using 10 CBCT scans on the study population of the same age group. Any discrepancy was eliminated from the main study by mutual consent. Every effort was taken to include a homogenous population in the study to eliminate bias.

The CBCT images were obtained using the three-dimensional (3D) Kodak CS 9300 CBCT machine with a tube voltage of 90 kV, tube current of 5 mA, exposure time of 11.30 s, and a cylindrical shaped field of view (FOV) measuring 17 mm × 13 mm with a voxel size of 300 *µ*. The FOV used was the same for all the scans to standardize the image selection criteria. All the images had a resolution of 90 *µ*m, and a single 360° scan was used. All the scans were taken with the patients standing upright in a natural head position and were instructed to contact their molars and breathe through their noses. The occlusal plane was positioned horizontally to the scan plane. The midsagittal plane was cantered. The planes on the three axes (*X*, *Y*, and *Z*) of the CBCT images were sequentially analyzed. The contrast and brightness of all the images were kept at a constant value for uniformity during image analyses. All images were assessed under optimal viewing conditions with appropriate image viewing software (Carestream 3D imaging software).

**Figure 1:**
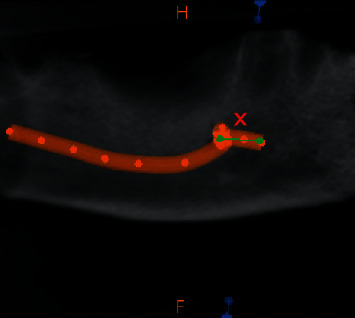
CBCT image showing anterior extension of alveolar nerve loop.

**Figure 2:**
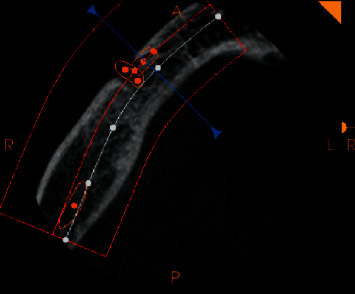
Measurement being carried out for the alveolar nerve loop using the measurement tool of standardized software.

On each volume, the axial slices were reconstructed parallel to the lower border of the mandible, and on the appropriate selected axial slice, the most anterior part of the mental foramen and the most anterior part of the inferior alveolar nerve was marked, and the length was measured. The length of the anterior loop was measured using the measuring tool of Carestream 3D imaging software(Figures 1 and 2). The measurements were carried out by two trained and calibrated observers. All the scans were measured and remeasured by the two observers until the desired inter and intraexaminer reliability was obtained (Cohen's kappa = 0.85).

All statistical analyses were performed using SPSS software v 20. Descriptive and analytical tests were performed. Frequency distribution analysis and chi-square tests were performed for categorical variables. ANOVA and independent sample *t*-tests were performed for comparison of mean length of anterior loop of the inferior alveolar nerve. *P* value < 0.05 was considered statistically significant.

## 3. Results

A total of 600 CBCT scans were evaluated. As regards age distribution, there was equal (33.3%) representation from all age groups. Also, 50% were males and 50% were dentate individuals who participated in the study. The population is homogenous in terms of age, gender, and dentition status with equal representation in all categories (Table 1).

An overall prevalence of 56.0% anterior nerve looping was found in the evaluated CBCT scans. The prevalence of anterior nerve looping on the left side was found to be 27.0%, whereas the prevalence of anterior nerve looping on the right side was found to be 29.0%. Although nonsignificant (*p* value > 0.05), the prevalence of the anterior nerve loop was found to be more on the right side when compared to the left side (Table 2).

Type 3 pattern of alveolar nerve loop was seen more commonly on right (48.0%) and left (47.8%) sides followed by type 1 and type 2 (Table 3).

A higher prevalence of anterior looping of the inferior alveolar nerve was seen in the age category of 20–40 years on both right (60.35%) and left sides (78.40%). As the age advanced, there was a significant decline in prevalence (*p* value <0.001^*∗*^) observed on both right and left sides. Males showed a significantly higher prevalence (*p* value < 0.005) of anterior looping of inferior alveolar nerves compared to females on both right (63.22%) and left sides (50.61%). No significant differences were observed (*p* value 0.89) between the partially edentulous and dentulous individuals on both right and left sides (Table 4).

The mean value of the anterior extension of the loop of the inferior alveolar nerve was evaluated, and it was found that there was a significant difference in age and gender. The mean value was significantly higher (*p* value <0.001^*∗*^) in the age group of 20–40 years on both right (1.61) and left sides (1.59), and there was a significant reduction in the mean length of the loop as the age advanced. The mean length was found to be significantly higher in males compared to females on both right (1.53) and left sides (1.39). No significant differences were observed in the mean length between dentate and partially edentulous individuals on both right and left sides (Table 5).

## 4. Discussion

The anterior loop of the inferior alveolar nerve is a sensitive anatomical feature that should be taken into account during the installation of dental implants anterior to the mental foramen [20]. With the advent of technology, there have been advancements in the field of surgical procedures and implants. The success of these surgical procedures and implants depends upon diagnostic acumens used for accurate identification of various nerves which needs to be preserved. Cone-beam computed tomography (CBCT) is an imaging modality with which such anatomical variants can be visualized and necessary modifications can be made during the presurgical phase of implant placement [21]. Anatomic variations in the mandible such as the anterior loop and lingual foramen can be better visualized in CBCT, thereby, preventing encounters of such variations during surgery [22]. Studies have shown that for accurate identification of the anterior loop CBCT is a better imaging modality when compared to panoramic radiography [12, 13, 17]. The present study evaluated the prevalence of anterior nerve looping and compared the mean values of anterior extension of the alveolar loop according to age, gender, and dentition status on the right side and left side using the advanced CBCT technique.

In the present study, the prevalence of the anterior loop of the inferior alveolar nerve was found to be 56.0%. This is similar to findings reported by Puri et al. [23] in their study carried out in Western India [23]. In a similar study carried out in the South Indian population, similar findings were reported by Sitaraman [21] On the contrary study carried out by Sinha et al. [24] in Eastern India population, it was found that the prevalence of anterior loop was only 9.7% [24]. There has been a wide variation observed in the prevalence of anterior loop as reported by several studies carried out in India and outside India, and the authors are of the opinion that these variations may be due to failure to accurately identify the loop, failure to use advanced diagnostic techniques for identification, or lack of training of investigator. Also, the criteria set for defining the IANAL could have resulted in high variation in its prevalence reported to date [25].

The prevalence of anterior looping inferior alveolar nerve was found to be more on the right side as compared to the left side. This is similar to the findings reported by Mishra et al. [26] in their systematic review and metanalysis and also by Sinha et al. [24] in their original research [24, 26]. In literature, there have been contrasting variations reported pertaining to side specificity of anterior loop of the inferior alveolar nerve. Some studies have reported that the prevalence of anterior loop is more on the left side compared to the right side [27]. Studies conducted by Puri [23] and Nascimento EHL et al. [10] found that there was no statistically significant difference between the right and left sides in terms of the presence of anterior loop [10, 23]. The authors are of the opinion that this variation may be due to varied ethnic and racial differences in a population.

Regarding the type of loop, it was found that type 3 was the most common followed by type 1 and type 2 on both the right and left sides. This is similar to findings reported by Prakash et al. and Shaban et al. [28, 29]. The abovementioned studies failed to evaluate the side-specific variations of the anterior loop of the inferior alveolar nerve. However, our study was more detailed and provided a complete picture of this variation on both right and left sides.

The prevalence of the anterior loop of the inferior alveolar nerve was found to vary significantly with age and gender. In the present study, it was found that as the age advanced, the prevalence of anterior loop reduced. This may be attributed to the fact that as age advances, the visibility of the alveolar nerve decreases. Similar findings have been reported by Sitaraman and Muthukrishnan and Uchida et al. [18, 21]. Also, the mean length of the anterior loop of the inferior alveolar nerve was found to decrease as the age advanced.

In the present study, males were found to have a higher prevalence of anterior loop of the inferior alveolar nerve compared to females. Also, males were found to have a higher mean length compared to females. Similar findings have been reported by Uchida Y et al., Sinha S et al,. and Sitaraman [18, 21, 24].

The average length of the loop as reported in the literature is between 0.4 and 6 mm [21]. In the present study, the mean length was found to vary from 1.14 to 1.61 which is similar to studies conducted by Apostolakis, Filo et al., and Watanbe et al. [4, 30, 31].

No significant variations were found with dentition status with prevalence or mean length of the anterior looping of the inferior alveolar nerve.

The study is unique in the sense that it was the first study carried out in the population of Lucknow, and the study findings will add to the existing data of the prevalence of anterior looping of inferior alveolar nerve carried out in the Indian population. A number of Indian studies have evaluated the prevalence of anterior loop but have failed to compare the variations on right and left sides and also the variations with regards to age, gender, and dentition status. Also, the variations in types of looping of the alveolar nerve loop have been rarely reported. This study has evaluated the prevalence of the anterior loop of the inferior alveolar nerve using the CBCT technique which is an advanced radiographic technique, and hence, the results obtained are more reliable. The study was carried out on a homogenous population in Lucknow with the aim to include equal age groups, equal gender, and equal individuals who were dentulous and partially edentulous. Every effort was taken to eliminate bias that might affect the outcome of the study. Also, unlike other Indian studies, the authors have evaluated nearly 550 scans which are ample to provide a complete picture of the prevalence of the nerve loop in the study population. The study included individuals from 20 to 80 years old and, thereby, provides a great opportunity to see the variations of the anterior loop of the inferior alveolar nerve with age. However, the generalization of the results can be questioned as this study was carried out in a single location. The authors recommend carrying out similar studies in multicentric locations before the results can be generalized.

## 5. Conclusion

The prevalence of the anterior loop of the inferior alveolar nerve was found to be 56%. The prevalence was found to be more on the right side compared to the left side. Type 3 anterior looping of the inferior alveolar nerve was more common followed by type 1. The prevalence of anterior looping and the mean length of the loop were found to vary significantly with age and gender. No significant variations were found with dentition status with prevalence or mean length of the anterior looping of the inferior alveolar nerve.

## Figures and Tables

**Table 1:** Demographic characteristics and dentition status of the study population.

Factor	Categories	Number	Percentage (%)
Age groups	20–40 years	200	33.33
	41–60 years	200	33.33
	61–80 years	200	33.33

Gender	Male	300	50.00
	Female	300	50.00

Dental status	Partially edentulous	300	50.00
	Dentate	300	50.00

**Table 2:** Prevalence of anterior nerve looping in the study population as obtained by the bilateral split of mandible into right and left halves.

Prevalence	No. (%)
Prevalence of anterior nerve looping on the left side (out of 1200 sides examined)	324 (27.0%)
Prevalence of anterior nerve looping on the right side (out of 1200 sides examined)	348 (29.0%)
Overall prevalence of anterior nerve looping (out of 2400 sides examined)	672 (56.0%)

**Table 3:** Frequency distribution of anterior nerve loop on right and left sides based upon their types.

Categories	Right side (out of 348 sides)	Left side (out of 324 sides)
Type 1	97 (27.9%)	94 (29.0%)
Type 2	84 (24.1%)	75 (23.2%)
Type 3	167 (48.0%)	155 (47.8%)

**Table 4:** Distribution of study population according to the prevalence of anterior looping of alveolar nerve on right and left sides.

Factor	Categories	Right side	Left side	*P* value (chi-square test)
Age	20–40 years	210 (60.35%)	254 (78.40%)	<0.001^*∗*^
	41–60 years	80 (22.99%)	40 (12.35%)	
	61–80 years	58 (16.67%)	30 (9.25%)	

Gender	Male	220 (63.22%)	164 (50.61%)	<0.001^*∗*^
	Female	128 (36.78%)	160 (49.39%)	

Dentition status	Partially edentulous	170 (48.85%)	160 (49.38%)	0.89
	Dentate	178 (51.15%)	164 (50.62%)	

^*∗*^*P* value < 0.05, statistically significant difference.

**Table 5:** Mean values of anterior extension of the alveolar loop on right and left sides according to age, gender, and dentition status.

Factor	Categories	Right side (mean ± SD)	*P* value	Left side (mean ± SD)	*P* value
Age	20–40 years	1.61 (0.37)	<0.001^*∗*^	1.59 (0.35)	<0.001^*∗*^
	41–60 years	1.53 (0.27)		1.40 (0.51)	
	61–80 years	1.23 (0.53)		1.14 (0.51)	

Gender	Male	1.53 (0.31)	<0.001^*∗*^	1.55 (0.32)	<0.001^*∗*^
	Female	1.39 (0.21)		1.43 (0.22)	

Dentition status	Partially edentulous	1.54 (0.31)	0.914	1.55 (0.32)	0.579
	Dentate	1.55 (0.27)		1.53 (0.27)	

^*∗*^*P* value < 0.05, statistically significant difference.

## Data Availability

The data used to support the findings of this study are available within the article and also from the corresponding author upon request.
